# ANN-Based Modeling of Engine Performance from Dynamometer Sensor Data

**DOI:** 10.3390/s26010120

**Published:** 2025-12-24

**Authors:** Constantin Lucian Aldea, Razvan Bocu, Rares Lucian Chiriac

**Affiliations:** Department of Mathematics and Computer Science, Transilvania University of Brasov, 500036 Brașov, Romania; costel.aldea@unitbv.ro (C.L.A.); rares-lucian.chiriac@unitbv.ro (R.L.C.)

**Keywords:** artificial neural network, internal combustion engine, sensor data analysis, engine load prediction, dynamometer measurements

## Abstract

Accurate prediction of the performance of an internal combustion engine is an essential step towards achieving efficiency and complying with emission standards. This study presents an artificial neural network (ANN) model that uses sensor-derived parameters, such as design power, wheel power, torque, and rotational speed, to predict engine load. Data were collected from a dynamometer and a hardware-in-the-loop (HiL) setup to ensure realistic, sensor-based measurements. The proposed ANN architecture achieved high accuracy (99%) in multiclass classification and strong regression performance ($R^{2}\approx 0.98$), demonstrating its ability to model complex engine load relationships under normal operating conditions. Performance was validated using 5-fold stratified cross-validation, achieving an average accuracy of 0.988±0.011, macro-F1 of 0.984±0.011, and regression $R^{2}$ of 0.962±0.052, confirming strong generalization and robustness. The model can be extended to include additional sensor inputs and adapted for use with other powertrain systems, allowing it to be used in a range of automotive and industrial applications.

## 1. Introduction

The collection and fine tuning of parameters, which relate to the operation of internal combustion engines, constitutes the object of several research projects. Thus, in [1], it is desired to develop an application from which the output value of the power of the internal combustion engine will be obtained. The objective of this paper was to predict the performance of the forecasting indicator with the input parameters spark timing, engine rotational speed, and load. The article [2] presented the possibility of using machine learning (ML) in artificial intelligence to classify the technical state of marine engine injectors. The technical condition of the internal combustion engine and injection apparatus significantly determine the composition of the outlet gases. The rest of the paper is structured according to the following sections. The second section introduces the most relevant similar existing solutions, while the third section analyzes the issues related to the measurement of collected data, which is followed by the external data that relates to the optimized models. The most significant aspects connected to the optimization of the proper machine learning models are discussed in the fifth section, which is followed by the conclusive remarks.

By using artificial neural networks and the data collected for training, the functional parameters of internal combustion engines are predicted, ensuring their efficient operation and compliance with regulatory standards without the need for extensive measurement.

The proposed methodology is demonstrated using a 1.9-L TDI light-duty diesel engine. However, it can be adapted to other engine types if sufficient sensor data and calibration parameters are available.

The paper [3] uses fuzzy logic (FL) and artificial neural networks (ANN) to predict in-cylinder pressure in a spark-ignition (SI) engine, based on experimental data. The engine was tested at various speeds and ignition timings. The ANN models achieved a high level of accuracy ($R^{2}>0.995$) and outperformed the FL models ($R^{2}\in \left[0.820,0.949\right]$). Furthermore, the trained artificial neural network model successfully predicted pressure and mean effective pressure (MEP) for conditions that had not been tested, producing results that closely matched the experimental data. The model also accurately identified the ignition timing for maximum brake torque (MBT), indicating its potential for engine optimization.

The modeling, diagnosis, optimization, and control of internal combustion engines through the consideration of modern machine learning techniques represents a very relevant field of study and research. Therefore, we have conducted a thorough literature review, which identified the most relevant technical solutions and research avenues. Thus, the article [4] reports a comprehensive scientific survey work. Thus, the article includes a critical evaluation of the existing Internal Combustion Engine (ICE) modeling paradigms. Additionally, it described optimization, diagnosis, and control challenges, together with promising state-of-the-art Machine Learning (ML) solutions. More specifically, certain major challenges that were identified include Real Driving Emission (RDE) modeling and control, combustion knock detection and control, combustion mode transition in multi-mode engines, combustion noise modeling and control, combustion instability and cyclic variability control. Furthermore, it is relevant to mention the financially expensive and time-consuming calibration of the engine, and also the related diagnosis processes, which pertain to certain ICE components. The paper approaches traditional ICE modeling solutions, while their limitations for real-time ICE optimization and control are assessed. Moreover, promising ML models that approach real-world ICE difficulties are classified considering three main groups of unsupervised learning, supervised learning, and reinforcement learning computational models. The working principles of each algorithmic model, which was evaluated, and also the implied advantages and disadvantages for the proper addressing of ICE challenges, are analyzed. It is relevant to note that particular ML-related gray-box solutions are suggested as adequate approaches, which blend the advantages that result from physics-based and ML-based models, to offer reliable and accurate solutions for the respective ICE modeling and control problems. Therefore, it may be stated that this survey article determines insight into the possible real-world applications of ML for ICE scenarios, while recommendations to address potential ICE challenges are provided.

There are interesting contributions that pertain to high-performance types of internal combustion engines. As an example, article [5] approaches the problematic of hybrid multi-mode machine learning-based fault diagnosis strategies with application to aircraft gas turbine engines. Thus, the authors reported a data-driven fault diagnostic framework, which is designed through the consideration of hybrid multi-mode machine learning strategies that are useful to monitor the system health status. The hybrid model that is based on multi-mode and concurrent faults, and also their adverse coupling effects, presents serious limitations for the specification of reliable diagnostic methodologies. Thus, the proposed solution considers the inherent embedded health information, which is determined by the input-output (I/O) sensor data. The efficiency of the reported solution is validated through the consideration of sensor data. This includes healthy samples, and also various structural and functional degradation modes, which relate to the compressor and turbine of aircraft gas turbine engines. Furthermore, the article describes a comparative analysis relative to other machine learning-based algorithmic models, which the authors consider to highlight the advantages of their solution. This specifically pertains to the accuracy of fault diagnosis, the rate of false alarms, and it also concerns the multi-mode and concurrent fault scenarios.

It is relevant to note that article [6] reports a contribution, which relates to the prediction of RCCI combustion fueled with CNG and algal biodiesel to sustain efficient diesel engines using machine learning algorithmic models. Thus, this paper considered microalgae biodiesel as a high-reactive fuel, which is directly injected along with various Compressed Natural Gas (CNG) energy shares: 10%, 20%, 30%, and 40%. The microalgae biodiesel is regarded as a low-reactive fuel supplied through the intake system. The experiments are conducted through a single-cylinder, water-cooled, 1500 rotations per minute, 3.5 kW power Compression Ignition (CI) engine. Thus, several loading conditions are considered to evaluate the effect of CNG energy share on the effective performance and emissions relative to the Reactivity Controlled Compression Ignition (RCCI) combustion mode. The outcomes of the experimental evaluation process suggest that the application of a 30% CNG share decreased Nitrogen oxides (NOx), and emitted smoke concentrations by 25% and 31%, respectively. Moreover, an increase in thermal efficiency of 4.35% was observed relative to traditional biodiesel combustion technologies. Last, but certainly not the least important, two machine learning models, more precisely the Gradient Boosting Regressor (GBR), and also LASSO (Least Absolute Shrinkage and Selection Operator) Regression, were designed and assessed in connection with the prediction of the individual dependent variables from the independent variables.

The solution reported in article [7] pertains to the machine learning assisted prediction of exhaust gas temperature of a heavy-duty natural gas spark ignition engine. The analysis relates to four different machine learning algorithms, more precisely the artificial neural network, random forest, support vector regression, and gradient boosting regression trees. If compared relative to one another, the four machine learning models predicted the exhaust gas temperature with an acceptable error, while also capturing its relationship with the three model inputs. The gradient boosting regression trees determined the optimal accuracy, but the drawback is represented by the necessity to consider noise-free data. Conversely, the random forest variant performed the worst in terms of accuracy, but it is also relatively tolerant to uncalibrated data. Additionally, it is relevant to note that the support vector regression determined the smallest error, but it required the highest amount of computational resources. Moreover, the artificial neural network algorithm was the optimal variation, but it is necessary to tune its hyperparameters. The conclusion of the study suggested that properly trained machine learning algorithmic models can semantically enrich relatively complex physical models, while also optimizing the engine’s performance, emissions, and lifespan.

Furthermore, paper [8] presents the limitations of conventional modeling and control strategies for internal combustion engines (ICEs), proposing machine learning (ML) as an alternative approach to addressing complex nonlinear behaviors. These reviews have highlighted several ongoing challenges, including compliance with real driving emissions (RDE), knock detection, transitions between combustion modes, cyclic variability, and the high cost of calibration and diagnostics. ML-based approaches, particularly artificial neural networks (ANNs), have been shown to overcome these issues by providing more accurate predictions than traditional methods and other soft computing techniques. ANN models successfully capture the subtle relationships between engine parameters, enabling reliable predictions of performance indicators under different operating conditions. Furthermore, ML solutions can be categorized into three paradigms: supervised, unsupervised, and reinforcement learning. This perspective aligns with recent findings showing that ANN-based frameworks outperform fuzzy logic and other models when it comes to predicting critical combustion metrics. This emphasizes their potential for real-time engine optimization and adaptive control.

The science of combustion represents an interdisciplinary field of study that includes nonlinear physical and chemical phenomena, which also relate to complex chemical reactions, and the study of fluid flows. Thus, paper [9] postulates that the improvement of the combustion processes is related to the enhancement of the efficiency with minimum emissions of pollution agents. Machine learning mediates the specification of data-driven techniques, which are considered for managing large amounts of combustion data that are acquired through either experimental processes or simulations that identify the hidden data patterns. This article presented an overview of contributions concerning the real-world usage scenarios of machine learning models relative to the scope of combustion science. Thus, this article intends to provide readers a description of the general scientific landscape, which relates to the utilization of machine learning in the scope of combustion research. Additionally, it is relevant to note that an interesting related scientific survey was presented in article [10].

Gasoline compression ignition (GCI) engines are regarded as an attractive alternative to traditional spark-ignition and diesel engines. The research that was reported in the article [11] relates to a Machine Learning-Grid Gradient Ascent (ML-GGA) solution, which was conceived to enhance the efficiency of internal combustion engines. Thus, machine learning models provide proper approaches to convert complex physical processes that take place in a combustion engine, into compact informational processes. Thus, the proposed ML-GGA solution was comparatively evaluated relative to a recent Machine Learning Genetic Algorithm. The article also provides specific technical descriptions of the considered procedures, optimization tools, and criteria, which should be considered to successfully attain the necessary levels of combustion engines’ efficiency.

The contribution that was reported in paper [12] relates to eleven regression models concerning properly chosen machine learning models. These were considered to generate a quick and accurate prediction regarding the start of combustion in homogeneous charge compression ignition engines, based on the usage of methane. The regression models determine a taxonomy concerning linear and nonlinear types. Although the described robust random sample consensus (RANSAC) model is a nonlinear type, in the same manner as SAM (simple algebraic model), the accuracy of the prediction was increased from 89.3% to 98.4%. This level of accuracy is attained relative to the linear models, more precisely, the ordinary least squares, ridge, and Bayesian ridge models. Considering the linear hypothesis, which relates to the correlation for the start of combustion prediction, the described algorithmic solutions reportedly generate a proper response time to be considered in real-time control applications, such as the electronic control units of the engines.

The contribution that was presented in article [13] interests a thorough survey regarding various real-world applications of ML models, considering a particular highlight on ANN that solves function approximation, optimization, monitoring, and control problems relative to the research of biodiesel. Furthermore, the advantages and disadvantages of using machine learning-based technologies in biodiesel research are targeted to calibrate upcoming research and development processes in the relevant domain. The fundamental justification for the consideration of machine learning models relative to the biodiesel industry regards the monitoring and control of biodiesel systems in a real-time manner. Nevertheless, these problems have seldom been explored in the relevant literature. Consequently, upcoming scientific studies may be calibrated towards the consideration of machine learning algorithmic models for the real-time processing, monitoring, and control of biodiesel systems. The essential goal of these efforts is to improve the efficiency of the production processes, along with the implied economic viability and environmental sustainability.

The goal of paper [14] is to analyze the possible specification of an AI/ML model, which may be proper for the predictive study and consequent design of an internal combustion engine. Thus, an artificial neural network (ANN) algorithm is selected as the algorithmic reference model, which describes and predicts the combustion process through the consideration of historical performance data produced using a computational model involving complex multiphysics flow dynamics, heat transfer, and chemical kinetics. Conceptually, multiphysics is defined as a coupled modeling approach of studies that demand simultaneous addressing of hitherto separate physical disciplines, and combining them to generate relational mathematical models and validate them with controlled experiments to enhance the understanding of natural behavior. Thus, the reported approach involves a series of key ANN parameters. For example, the number of hidden layers, number of neurons, and activation functions are numerically experimented to achieve an adequate level of prediction accuracy through the minimization of the losses that are induced by the training and validation processes. It is relevant to note that article [15] described an interesting related algorithmic model, which studies the effects of injection timing, injection pressure, and exhaust gas recirculation rate on the combustion processes that occur inside the engines.

Machine learning techniques have been widely applied to modeling internal combustion engines, but there are still gaps in the current literature. Most studies rely exclusively on simulation data or dynamometer measurements without using hardware-in-the-loop (HiL) testing to replicate real-world operating conditions. Additionally, datasets containing essential parameters, such as design power, wheel power, torque, and rotational speed, are scarce and publicly available datasets are even scarcer. This limits reproducibility and benchmarking. Existing approaches tend to focus on narrow operational scenarios and often overlook the potential for real-time monitoring and predictive maintenance. This work addresses these gaps by combining dynamometer and HiL data to estimate loads, publishing a curated dataset containing key variables, and demonstrating an artificial neural network (ANN)-based methodology that reduces calibration effort while enabling real-time predictive capabilities.

The chassis dynamometer provides calibrated and repeatable ground-truth labels for load through measured wheel power and torque under controlled sweep conditions. This ensures high label fidelity and realistic measurement noise. The HiL setup complements this by exercising the ECU-sensor-actuator loop in real time. It allows for the safe variation of operating scenarios, such as transients, accelerations/decelerations, and ambient changes. It also captures control-system behavior that does not appear in static dyno runs. Training an ANN with data from both sources yields a model that is (i) physically calibrated by dyno measurements, (ii) ready for deployment because its inputs and outputs mirror the signals available to the ECU, and (iii) more robust in real operating conditions. In practice, this reduces calibration effort, improves generalization across regimes, and simplifies integration of the trained model into embedded or cloud monitoring pipelines.

The contributions of this study can be summarized as follows:

(1) We propose a framework based on artificial neural networks (ANNs) for predicting engine load using sensor-derived parameters such as design power, wheel power, torque, and rotational speed. By using this kind of approach the effort and data requirements for calibration could be reduced.

(2) The proposed model demonstrates high predictive performance. It achieves 99% accuracy in multiclass classification and has strong regression capabilities ($R^{2}\approx 0.98$), which confirms its effectiveness in capturing non-linear dependencies among engine parameters.

(3) Unlike previous ANN-based approaches that primarily relied on simulation data or isolated dynamometer measurements, dynamometer and hardware-in-the-loop (HiL) data were combined in this study to capture realistic operating conditions for engine load estimation.

(4) Leveraging historical performance data enables the approach to provide real-time monitoring and predictive maintenance, addressing practical challenges that prior literature has not fully resolved.

(5) This paper discusses how adaptable the proposed architecture is to other powertrain systems, emphasizing its potential for use in a wider range of applications in the automotive and industrial sectors.

## 2. Issues Related to the Measurement of Collected Data

**Figure 1:**
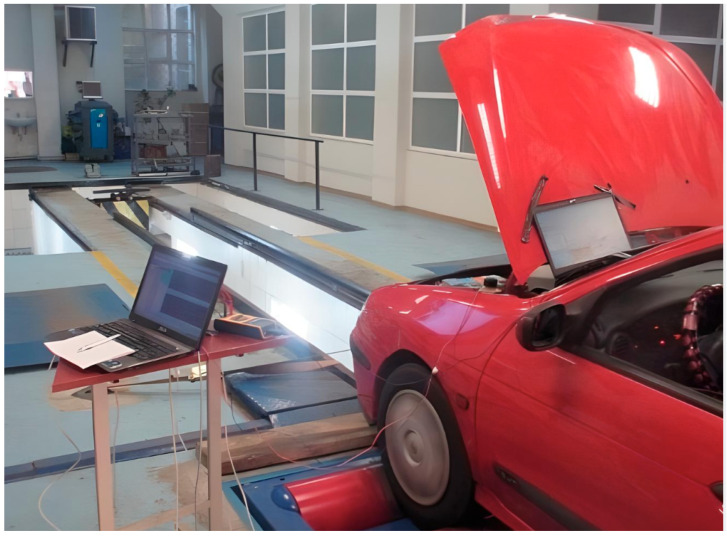
Experiment for data retrieval and collection [16].

Both experimental setups and virtual simulations were used to take the measurements described in [16] (Figure 1).

The experimental measurements were taken using a physically constructed hybrid turbocharger and a CIMAT Turbo Test Pro stand. The latter simulates exhaust gas flow using compressed air at a pressure of between 0.1 and 0.2 MPa. The key parameters measured included turbocharger shaft speed (up to 160,000 rpm); electric generator shaft speed (14,000–16,000 rpm via a 1:10 gear ratio); voltage (0–23 V); current (0–4.5 A); power output (up to 115 W). Measurements were taken using analog devices and data acquisition systems.

The virtual simulations involved modeling and simulating the system using AMESim software by Siemens. The simulations included: turbocharger rotor speed and compressor outlet pressure over time; electrical power generation based on thermodynamic equations; comparative performance analysis between naturally aspirated and hybrid turbocharged engines.

The dataset [17] used in this paper was not initially collected as it was intended to be analyzed further in this article.

One of the problems related to data retrieval could be the accuracy of data processing. There are chances that faults in data collection exist, and the data need to be corrected using outlier detection and removal methods.

The MAHA LPS 3000 dynamometer that we used for the experimental results is frequently utilized in both scientific and engineering research, particularly for analyzing fuel efficiency, emissions, and the performance of internal combustion engines under varying conditions [18]. This system provides highly accurate measurements of vehicle power and torque, which makes it ideal for experimental setups that need to simulate realistic driving conditions, like acceleration, high-load scenarios, and different terrain resistances. The LPS 3000 can precisely measure design power (in kW or HP) and maximum engine torque (in Nm) through its roller system. This model typically supports measurements up to approximately 1100 HP, which is sufficient for most production vehicles as well as many high-performance cars. The LPS 3000 supports a maximum testing speed of up to 70,000 RPM, making it suitable for high-speed vehicles and advanced performance testing. The LPS 3000 features specialized software called PowerDyno, which visualizes and records data in real time, displaying parameters such as engine rotational speed, power, torque, and more. This software enables the generation of detailed reports and comparison of data across various tests, which is highly beneficial in tuning and advanced diagnostics. The dynamometer allows for calibration to ensure measurement accuracy and is certified according to European standards. This makes it appropriate for use in vehicle inspection centers and for verifying vehicle compliance with emissions and performance standards [19].

The MAHA dynamometer, such as the LPS 3000, is designed to test vehicle performance by measuring critical parameters like design power, torque, and various driving conditions. Here’s a breakdown of its input data, output data, and sensors involved in its functioning, which is briefly given.

Input data for a dynamometer generally includes:

- Engine Rotational Speed: This is provided by the vehicle’s control systems or sensors and is essential for calculating power and torque.
- Environmental Parameters: Inputs such as ambient temperature, air pressure, and humidity are recorded to account for their impact on engine performance.
- Vehicle Load Data: For more realistic testing, user-defined load simulations may be configured to replicate different driving conditions, including gradients or aerodynamic drag.

MAHA dynamometers are equipped with various high-precision sensors:

- Rotational Speed Sensors: These sensors measure the roller speed to calculate engine rotational speed and, in turn, help derive torque and power.
- Load Cells: These measure the force exerted on the rollers by the drive wheels, crucial for torque calculation.
- Temperature Sensors: These monitor the roller temperature and sometimes the exhaust temperature to ensure accurate data and prevent overheating.
- Pressure Sensors: Integrated in emissions testing models to monitor and adjust for atmospheric pressure.

The MAHA dynamometer relies on regular calibration to maintain precision, often to within ±1%, as noted in professional automotive testing standards. This accuracy is crucial for applications in research, tuning, and compliance testing in the automotive industry, providing consistent results by compensating for factors like tire slip or environmental variation.

Overall, the MAHA dynamometer stands out due to its high-precision sensors, customizable testing parameters, and robust software integration, making it a reliable choice for both performance tuning and technical diagnostics [20,21].

## 3. Measurement Apparatus

Design power, often denoted as $P_{n}$, refers to the engine’s rated or standard output power under specific operating conditions, usually when running at its maximum efficiency. This is the power the engine is designed to deliver under normal conditions (without overstressing the components). Design power is often specified by the manufacturer and is used to represent the engine’s performance in general operating scenarios. In automotive terms, this is the maximum power the engine can deliver at a specific *n*, typically at high revolutions per minute (rpm). For example, in cars, design power might be achieved at around 3000 to 6000 RPM depending on the engine design [22,23].

(1) $$P_{n}=\frac{M_{n}\cdot n}{9.5488\cdot 10^{3}}$$

where:

- $P_{n}$ is design power (measured in kW).
- $M_{n}$ is the engine torque (in Nm).
- *n* is the engine rotational speed in revolutions per minute (RPM).
- The constant 9.5488 converts the torque-speed product to kilowatts (kW), based on the relationship between power, torque, and engine rotational speed.

Wheel power, denoted as $P_{wheel}$, is the actual power that is transmitted to the wheels of the vehicle. It is lower than the engine’s design power due to losses in the drivetrain, which includes components like the transmission, differential, and other mechanical parts that transfer power from the engine to the wheels [24]. These losses are often referred to as mechanical losses. In practice, the wheel power is measured at the wheels and is the effective power available for the movement of the vehicle. A car’s engine might produce, say, 200 kW of design power, but after accounting for drivetrain losses (typically 15–20%), the wheel power might only be around 160 kW [25].

(2) $$P_{wheel}=\eta_{dt}\cdot P_{n}=\left(1-\delta_{dt}\right)\cdot P_{n}$$

where:

- $P_{n}$ is nominal or design power (measured in kW).
- $\eta_{dt}$ is drivetrain efficiency and $\delta_{dt}$ is drivetrain loss percentage and represents the proportion of power lost in the drivetrain, typically ranging from 10% to 20% for many vehicles. For example, if drivetrain losses are 15%, then $\delta_{dt}=0.15$, and then $\eta_{dt}=0.85$. Drivetrain loss percentage in (2) is derived from MAHA LPS 3000 outputs (wheel power, drag power, and standardized design power extrapolation). The MAHA software computes parasitic losses of the dynamometer, vehicle drivetrain, and tire-roller interaction; the percentage is calculated as $\left(P_{n}-P_{wheel}\right)/P_{n}$.

Engine torque, $M_{n}$ measured in newton-meters (Nm) or kilonewton-meters (kNm), but it can also be expressed in kW if calculating torque as a function of power and engine rotational speed. Engine torque (also known as nominal torque) refers to the engine’s rated torque output at a specific engine rotational speed (typically at a certain engine speed in RPM). It’s the force that the engine applies at the crankshaft to rotate the vehicle’s components. Torque is a key factor in determining the engine’s ability to do work. Relationship to Power (*P*) and torque (*M*) are related by the Equation (3).

(3) $$M_{n}=\frac{P_{n}\cdot \left(9.5488\cdot 10^{3}\right)}{n}$$

where:

- $M_{n}$ is the nominal moment or engine torque or crankshaft torque measured in Nm. Conversion to kW occurs only when using Equation (3) for power calculations.
- $P_{n}$ is the design power in kW.
- *n* is the engine rotational speed in RPM.
- The constant 9.5488 adjusts for the unit conversion between kW, Nm, and RPM [26].

Engine rotational speed, symbolized as *n*, describes the engine’s crankshaft which rotates and is measured in revolutions per minute (RPM). The engine rotational speed is critical because it determines both the power and torque characteristics of the engine at any given moment. Combustion engines generally have a broad range of speeds at which they operate efficiently. Low speeds (around idle RPM) usually correspond to low power and torque, while high engine rotational speeds (at or near the engine’s redline) correspond to peak power outputs. Engine rotational speed is inversely related to torque in many combustion engines — higher *n* generally results in lower torque, while lower *n* yields higher torque [27].

Design power ($P_{n}$) depends on torque ($M_{n}$) and engine rotational speed (*n*). Wheel power ($P_{wheel}$) is lower than design power due to drivetrain losses. Engine torque ($M_{n}$) and engine rotational speed (*n*) are related: higher engine rotational speed typically results in lower torque, but power can still be high at higher engine speeds (*n*). The torque at the wheels is typically lower than engine torque because of drivetrain losses, and wheel power reflects this reduction.

Understanding these relationships helps to evaluate engine performance, fuel efficiency, and how power is delivered to the vehicle’s wheels for various driving conditions [28].

The 1.9 L TDI diesel engine used was a reliable and efficient option in the mid-size and compact car market. It offered strong performance, good fuel economy, and impressive durability, making it a popular choice for drivers looking for a practical vehicle that could provide long-term service.

This engine provided a good balance of power and fuel economy, making it a favorite among consumers who needed a daily commuter car with solid performance and low running costs.

The 1.9-L TDI diesel engine has a displacement of 1896 cc and delivers a power output of 65 kW (or 89 PS). It features a robust inline-4 configuration, using diesel as its fuel type. This engine incorporates Turbocharged Direct Injection (TDI) technology, utilizing either the Common Rail or Pumpe Düse (PD) injection systems, depending on the model variation. To enhance performance, it is equipped with a turbocharger that increases air intake, boosting the engine’s overall power output. Its cooling system relies on water cooling, often complemented by an intercooler, which helps cool the compressed air from the turbocharger, further improving efficiency and maintaining optimal performance. The TDI engine used a turbocharger to force more air into the engine for combustion. This allows for more power from a smaller engine displacement. The intercooler reduced the temperature of the compressed air from the turbo, improving efficiency and performance. The engine utilized direct fuel injection to deliver fuel straight into the combustion chamber [29,30]. This method offers better fuel efficiency, more precise control of the combustion process, and lower emissions compared to older carbureted or indirect injection systems. 1.9 TDI engines used Variable Valve Timing (VVT) to optimize the engine’s performance at different RPM ranges. This helped in improving fuel efficiency and reducing emissions [31]. Over time, the turbocharger could suffer from wear, leading to issues like reduced power output, increased smoke from the exhaust, or a loss of turbo boost. Regular oil changes were critical to prolonging turbo life [32]. Thanks to the TDI technology, the engine was very fuel-efficient, with average fuel consumption of around 5.5 to 6.5 L/100 km [33].

The input parameters used to train the model are: design power ($P_{n}$ measured in [kW]), wheel power ($P_{wheel}$ measured in [kW]), engine torque ($M_{n}$ measured in [Nm]), engine rotational speed (*n* measured in [RPM]).

The design power is typically measured by placing the engine on a dynamometer. This machine applies a load to the engine and records the power output at a specific *n*, usually around the engine’s rated *n*. These values are taken under controlled conditions, often simulating standard driving or operating conditions. This is the actual power delivered to the wheels of the vehicle, which is measured after accounting for drivetrain losses (such as transmission, differential, and other mechanical components). A chassis dynamometer is typically used, where the vehicle is driven onto rollers, and power at the wheels is measured while the car operates under various loads and engine rotational speeds. Torque is measured in a similar manner, using a torque sensor or dynamometer to determine the torque applied at the engine’s crankshaft at a given *n*. The engine rotational speed, measured in revolutions per minute (RPM), is recorded during testing. This can be done using a tachometer or by reading from the vehicle’s ECU (Engine Control Unit). The speed data allows for the correlation between torque, power, and the rotational speed of the engine [34].

The HiL (Hardware-in-the-Loop) setup provides a real-time testing environment where the actual engine control unit (ECU) and hardware are interfaced with software simulations that replicate different operating conditions. HiL testing enables engineers to evaluate how the engine responds under a variety of conditions such as acceleration, deceleration, different loads, and varying environmental factors [35].

Using HiL, all data related to $P_{n}$, $P_{wheel}$, $M_{n}$, and *n* are logged in real-time. This data can then be analyzed to observe trends, test various control algorithms, and optimize engine calibration for fuel efficiency, emissions, and power delivery. High-fidelity models allow engineers to study the impact of changes in the engine parameters on overall vehicle performance [36].

HiL setups often include components such as sensors, actuators, and ECUs which interact directly with physical systems in the vehicle. These components provide real-time feedback to the control systems and allow for the fine-tuning of parameters like fuel injection, turbocharging, and exhaust recirculation, all of which affect power, torque, and efficiency. This setup helps in gathering experimental data that reflects real-world behavior [37].

Sample data for measured functional parameters as shown in the Table 1 were collected for an engine load of 0.25, 0.50, 0.75 and 1.00, respectively [17].

This dataset, Table 2, is well-balanced across load classes (The dataset contains 483 samples for Load = 0.25, 523 samples for Load = 0.50, 527 samples for Load = 0.75, and 531 samples for Load = 1.00) and provides a set of continuous features suitable for both regression and classification tasks.

**Figure 2:**
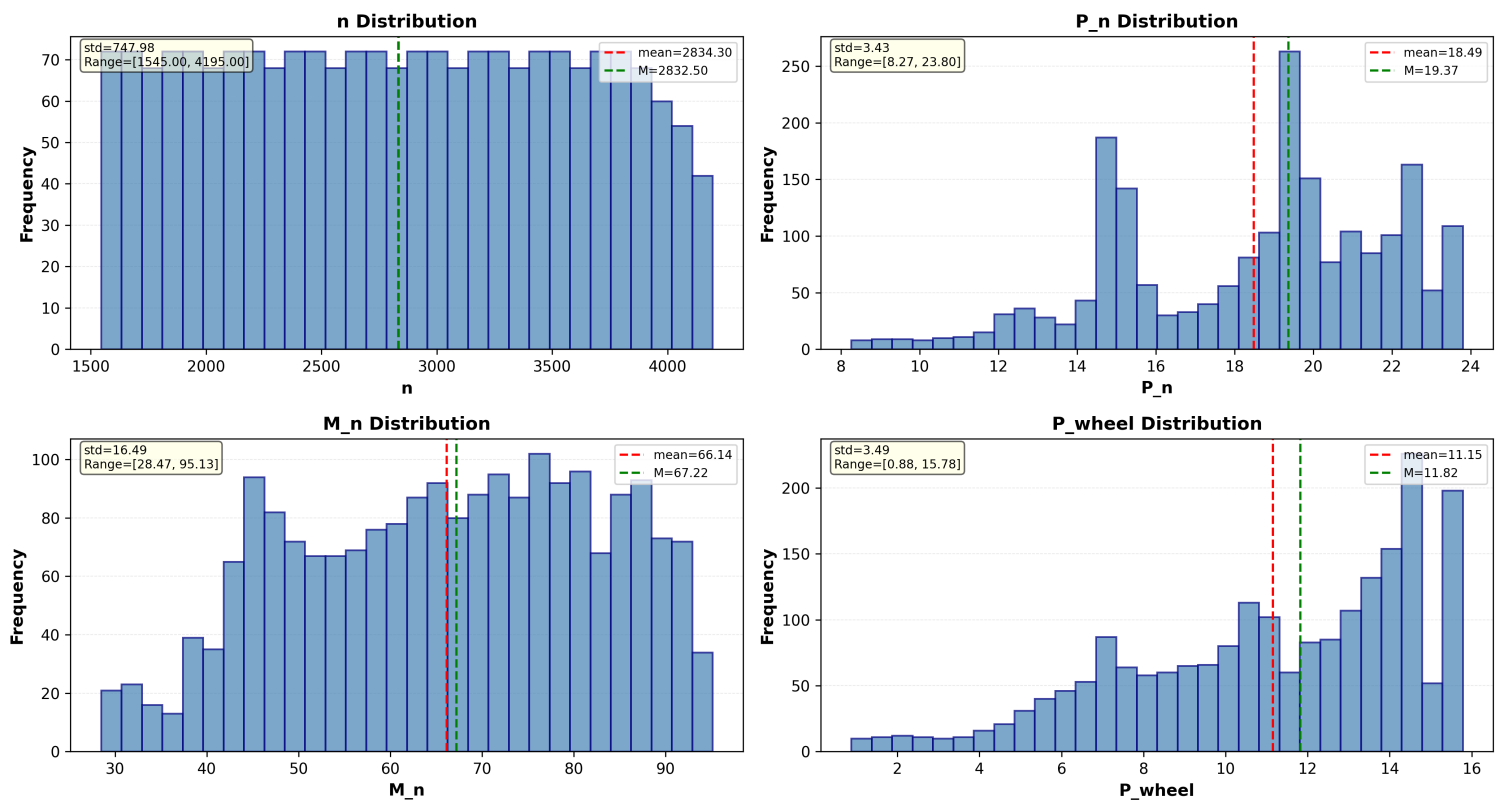
Dataset Overview.

The distribution of the values of the analysed features across the input dataset is shown in Figure 2. The Engine Rotational Speed values are evenly distributed throughout their range, suggesting that there is no specific area of high concentration. The design power has several common value ranges, indicating the presence of distinct operating modes or clusters in the data. The torque values form a bell-shaped curve, meaning they tend to cluster around a central value. There is a tendency for wheel power to increase across the range, which may indicate a bias towards higher power values or a process that ramps up over time.

Other external factors that may influence the models include ambient temperature and the cetane number of the diesel fuel.

This study used a dataset comprising 2064 samples collected across four discrete load classes (0.25, 0.50, 0.75 and 1.00), with 483, 523, 527 and 531 samples in each class, respectively. The engine speed ranges from 1545 to 4195 rpm and is recorded in quasi-steady increments of 5 rpm within continuous sweeps at each load level. During model training, all continuous features were scaled using a standardizer with a zero mean and unit variance to prevent large-range variables from dominating. Outlier screening was performed using the interquartile range (IQR) criterion (Q1−1.5 × IQR and Q3 + 1.5 × IQR) for each continuous variable. Each load class was acquired by sweeping the engine’s rotational speed range almost continuously, in increments of 5 RPM.

## 4. Experimental Evaluation Relative to Reference Machine Learning Models

Classification in Machine Learning is a supervised task, where the goal is to predict the type of an object based on its features. We distinguish between binary and multiclass classification. In binary classification, the goal is to sort data into two distinct categories, while in multi-class classification, there are more than two categories available for prediction. However, at their core, the two classification problems are linked to probabilities: typically, an output of a ML classification algorithm is a set of probabilities that is then translated into the actual prediction.

Different algorithms are available for classification, roughly categorized by the type of boundary they draw between the available classes. We have linear, quadratic, and non-linear classifiers, each with its own advantages and disadvantages. A key benefit of linear classifiers is that they are easy to interpret. They are fairly limited by their linear decision boundary. Most complex problems do not allow the data to be linearly separable, meaning that simpler linear models lose their applicability.

We therefore focus on non-linear classifiers, more specifically artificial neural networks. As their name suggests, they are inspired by the function and structure of the biological neural network. The unit in such a network is the perceptron, which takes as input the features of the considered object. These features are assigned different weights, after which an activation function is applied. Activation functions decide whether a neuron should be activated. During training, our aim is to learn the weights and biases in order to reach as close as possible to the ground truth.

On its own, a perceptron is a binary linear classifier. In a very simple network consisting of only one perceptron, the activation function is the step function. This means that the simple network will output whether the object is classified as a specific class. However, artificial neural networks consist of far more than a single perceptron.

In an ANN, we distinguish between three types of layers: input layer, a variable number of hidden layers, and the output layer. Depending on the task performed by the ANN or the way loss is defined, the activation functions for the hidden layer and the output layer may differ. For example, in a multiclass classification problem we may choose ReLu as an activation function for the hidden layer to facilitate communication between the nodes and softmax activation for the output layer, as here we are interested in the class to which the input object most likely belongs.

A network is typically called a deep neural network if it has at least two hidden layers. In the majority of neural networks, two adjacent layers are fully connected with each other. As mentioned, the importance of each neuron for classification is determined by the activation function used.

Training an artificial neural network has two steps: the forward pass and the backwards pass. The core idea behind it is to optimize the networks performance by minimizing the difference between the predicted output and the actual target values. This is known as empirical risk minimization. We do this by adjusting the weights and biases in the network (backward pass) based on the computed loss (forward pass).

Although they are both types of supervised learning techniques used in machine learning and statistics, classification predicts a category while regression predicts a continuous output value.

**Figure 3:**
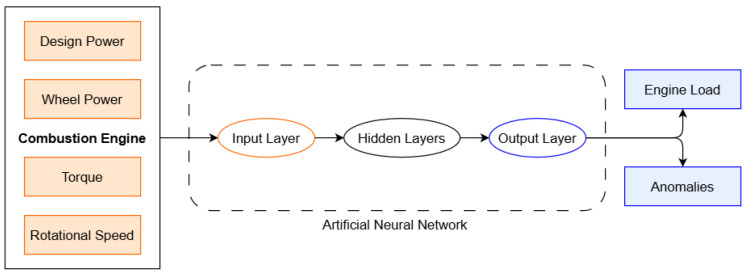
ANN architecture for non binary classification.

The current paper analyzes and proposes a concept for using artificial neural networks to predict engine load based on the input parameters mentioned above (design power, wheel power, engine torque, and engine rotational speed). The ANN architecture shown in Figure 3 uses the data measured by the authors. This architecture can be easily extended to use a larger number of input parameters and to perform more complex classifications or optimizations of the working parameters of an engine. The model comprises four layers: an input layer that takes a 4-dimensional feature vector (4); three hidden layers which apply nonlinear transformations using activation functions; and an output layer with a single neuron with no activation or a linear activation for regression. The output layer produces class scores, which are converted into probabilities using the softmax function. The model learns a non-linear mapping from a four-dimensional input to a probability distribution across four categories. Backpropagation and an optimizer (e.g., Adam) are used to learn the weights and biases during training.

Classification Model

The classification task predicts discrete engine load classes (0.25, 0.50, 0.75, 1.00) from four sensor-derived features: design power ($P_{n}$), wheel power ($P_{wheel}$), torque ($M_{n}$), and rotational speed (*n*). The architecture and training configuration are as follows:
Input Layer4 neurons (one per feature)Hidden Layers-Dense (30, activation = ReLU) + Dropout (0.2)-Dense (18, activation = ReLU) + Dropout (0.1)Output LayerDense (4, activation = Softmax)Loss FunctionSparse categorical cross-entropyOptimizerAdam (learning rate $10^{-3}$)Training SettingsBatch size = 10, epochs = 100, early stopping (patience = 15, min_delta = $10^{-5}$)RegularizationDropout only, no L2 or batch normalizationValidationStratified train/test split (70/30), random seed = 12, and features scaled to zero mean and unit variance.

**Figure 4:**
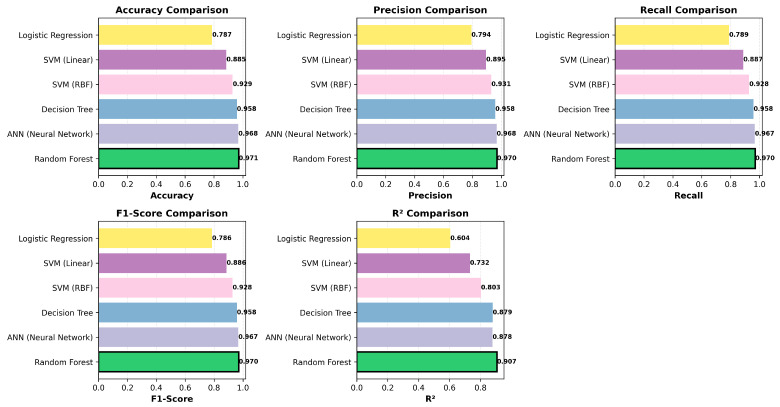
Performance comparison of ANN with baseline ML models.

In the Figure 4, the comparison of the ANN model with baseline models highlights the superior performance of the proposed ANN model over traditional machine learning baselines. While logistic regression and decision trees achieved moderate accuracy (approximately 0.79 and 0.85, respectively) and low $R^{2}$ values (0.604 and 0.878), the ANN consistently delivered higher predictive capability across all metrics. Specifically, the ANN achieved the highest $R^{2}$ (approximately 0.878) and competitive classification scores, outperforming SVM variants and random forest in precision and recall stability. Random forest showed strong regression performance ($R^{2}\approx 0.907$) but lagged behind in classification metrics compared to ANN. These results confirm that the ANN architecture effectively captures tendencies present in engine data. This architecture offers a balanced set of benefits for both classification and regression tasks.

The model achieved an accuracy of approximately 0.99 and macro-F1 score of 0.99. The confusion matrix shows less than 3% misclassification, confirming strong generalization.

Regression Model

The regression task involves predicting continuous engine load values under the same input conditions. The architecture is similar to the classification model, except for the output layer:
Input Layer4 neuronsHidden Layers(same as classification)
-Dense (30, activation = ReLU) + Dropout (0.2)-Dense (18, activation = ReLU) + Dropout (0.1)Output LayerDense (1, activation = Linear)Loss FunctionMean squared error (MSE)OptimizerAdamTraining Settings(same as classification) Batch size = 10, epochs = 100, early stopping (patience = 15, min_delta = $10^{-5}$).

To ensure methodological transparency and reproducibility, Table 3 summarizes the key hyperparameters used for the classification and regression models. These include architectural details, such as the number of neurons per layer and activation functions; optimization settings, such as the learning rate and optimizer type; and training configurations, such as the batch size, number of epochs, and early stopping criteria. Regularization strategies, including dropout rates, are also reported. This comparison highlights the consistency in training protocols while noting the differences in output layer design and loss functions.

**Figure 5:**
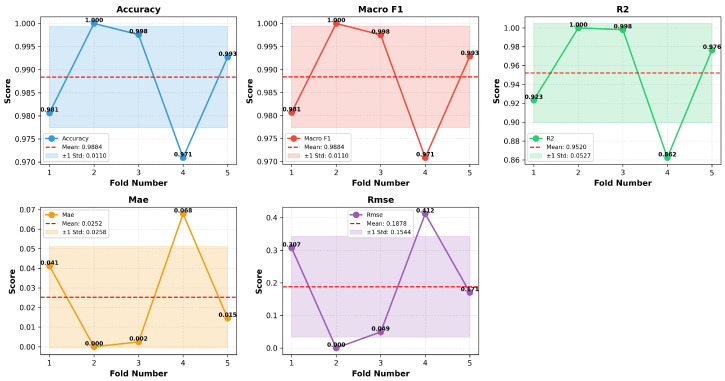
5-fold Stratified Cross-Validation.

The 5-fold stratified cross-validation, from the Figure 5, demonstrates strong and consistent performance across all folds for both classification and regression tasks. Classification metrics show near-perfect stability, with accuracy averaging 0.988±0.011 and macro-F1 at 0.984±0.011, indicating that the model generalizes well and maintains class balance across folds. Regression performance is similarly robust, with $R^{2}$ averaging 0.962±0.052, confirming the model’s ability to describe the correlation between input features and engine load. Error metrics remain low, with MAE at 0.027±0.028 and RMSE at 0.187±0.195, though fold-to-fold variability suggests occasional sensitivity to specific data partitions. Overall, these results validate the reliability of the ANN architecture and confirm that the proposed approach achieves high predictive accuracy with minimal variance, reinforcing its suitability for real-world deployment in engine performance monitoring.

In the case of classification problems, a neural network uses a function to map the input features onto a probability distribution for the discrete classes. This can be expressed as

$$f:R^{n}\to \Delta^{C-1}.$$

where:
$R^{n}$is the input space (e.g., n=4 for four features)$\Delta^{C-1}$is the (C−1)-dimensional probability simplex, representing a probability distribution over *C* classes.

The regression is a function $f:R^{4}\to R$ that maps the input feature $x\in R^{4}$ to a continuous image y∈R.

By replacing the softmax activation with a linear activation (or no activation) and setting the number of output neurons to 1 (or more if you are predicting multiple continuous values), regression instead of classification is obtained.

Let us denote the components of a feedforward neural network as follows:
$x\in R^{n}$input vector with *n* features$W^{\left(l\right)}$weight matrix of layer *l*$b^{\left(l\right)}$bias vector of layer *l*$z^{\left(l\right)}$pre-activation output of layer *l*$a^{\left(l\right)}$activation output of layer *l*

Let the input vector be:

(4) $$x=\left(\begin{array}{c} x_{1}\\ x_{2}\\ x_{3}\\ x_{4} \end{array}\right)=\left(\begin{array}{c} \mathrm{Design}\;\mathrm{Power}\\ \mathrm{Wheel}\;\mathrm{Power}\\ \mathrm{Engine}\;\mathrm{Torque}\;\\ \mathrm{Engine}\;\mathrm{rotational}\;\mathrm{speed} \end{array}\right)\in R^{4}$$

ANN layer details are in Appendix Table A1, and the architectures were evaluated as follows.

1. Data preparation. In this phase, the sensor data were prepared as input for the ANN under consideration. The input data used to train the artificial neural networks were preprocessed, and the outliers were removed from the acquired data. A cleaned version was then published online [17].

2. Prepare the training and test data. Within this phase, the data were randomly divided into two parts: training data (0.7) and test data for model validation (0.3). The input data were transformed using the standard scaler. Scaling prevents features (the input parameters) with larger ranges from dominating the learning and ensures that all features contribute equally to model training. Since the proposed model will predict multiple load classes, the input data must be converted to the corresponding class (class 1 for 0.25 loads, class 2 for 0.5 loads, class 3 for 0.75 loads, and class 4 for 1 load) in order to utilize the loss function for multiclass classification problems, in which each sample belongs to exactly one class. (*sparse_categorical_crossentropy*).

3. Model creation. A sequential ANN model with an architecture consisting of an input layer with the number of neurons equal to the number of input parameters, multiple hidden layers (3, 5, 8, AND 10 LAYERS) and an output layer was designed, implemented, compiled, and evaluated. The number of neurons in the hidden layer was adjusted to obtain better metrics. The activation function used is a ReLU (Rectified Linear Unit) on the hidden layers, due to its efficiency. The Softmax function was used as the activation function for the output layer, meaning that the input with the highest value would have the highest probability, while still allowing all inputs to influence the result. The sparse cross-entropy loss function was used to measure the performance of the model by comparing the predicted class probabilities to the actual class. The network can learn complex patterns using activation functions.

ANN variants of increasing depth were evaluated using binary cross-entropy, and no accuracy gains were found with more hidden layers. Training time increased due to the larger parameter count and the additional backpropagation steps required. Similarly, the correct/incorrect tallies indicated poor performance, confirming that this type of model is not suitable for this task.

To evaluate the effectiveness of the proposed artificial neural network (ANN) architecture, we conducted regression and classification analyzes using binary classification. The regression metrics showed that the 5-layer model had the highest predictive accuracy, achieving the greatest number of correct predictions (581) and the lowest error rate (39), while maintaining a competitive binary cross-entropy loss of 0.47.

Binary classification was not suitable for the current problem. Engine load prediction involves multiple discrete categories rather than just two states. Therefore, the problem was reformulated as a multiclass classification task. The model achieved 0.99 accuracy using multiclass classification.

4. Model training. The model was trained for 100 epochs and the model with the maximum accuracy value was selected. Hyperparameters such as number of epochs, network architecture, learning rate and batch size variations are used to improve the performance of the model.

The model was then saved and reloaded when needed to make predictions.

The model can be further discussed and improved. Although the model performs well, the accuracy can be improved. Accuracy can be improved by increasing the input dataset, having a good distribution of data classes, creation and analysis of more complex models with advanced optimizers, avoiding overfitting and underfitting. In this case, taking into account the measured data, an increase in the number of hidden layers of the ANN does not lead to an improvement of the model accuracy.

5. Make predictions. Using the test data, the model was evaluated, and the number of correct predictions was counted. Also, for manually selected sample data (Table 4), the predictions are made for the motor load values, and the values are in the appropriate interval from the input training set.

When the predicted values are continuous (see Table 4), the deep neural networks are used for regression to predict values in the input conditions. To transform these values into a category for classification purposes, the predicted values are approximated to the closest category. In this sense, the current paper employs the regression model for making predictions as a classification method. Nevertheless, if exact predicted values are required, the proposed proof of concept can provide these by using categorical cross-entropy as a multiclass loss function to generate label-based values.

As can be seen (Table 5), the $P_{n}$ exhibits the strongest linear relationship with the predicted load ($R^{2}\approx 0.87$). *n* also shows a strong correlation ($R^{2}\approx 0.72$). In contrast, the $M_{n}$ and $P_{wheel}$ demonstrate weaker linear relationships, suggesting that other models or transformations might be more effective in capturing their influence.

The $R^{2}$ values presented in Table 6 shows that certain variables, such as torque ($M_{n}$) and wheel power ($P_{wheel}$), exhibit relatively low coefficients of determination compared to design power ($P_{n}$) and engine rotational speed *n*. This indicates that their relationship with engine load is weakly linear and more complex in nature. While polynomial regression improves the fit slightly, these features are better modeled through non-linear approaches such as artificial neural networks, which can capture multidimensional dependencies among parameters. The observed variation in $R^{2}$ values therefore reinforces the need for advanced models like ANN for accurate load prediction, rather than relying solely on simple linear or polynomial regressions.

In the case of Polynomial Regression, in the Table 6, $P_{n}$ shows a significant improvement with polynomial regression, especially at degree 3. The *n* also benefits from a higher-degree model. Even though $M_{n}$ and $P_{wheel}$ show slight improvements, they still provide relatively weak fits.

**Figure 6:**
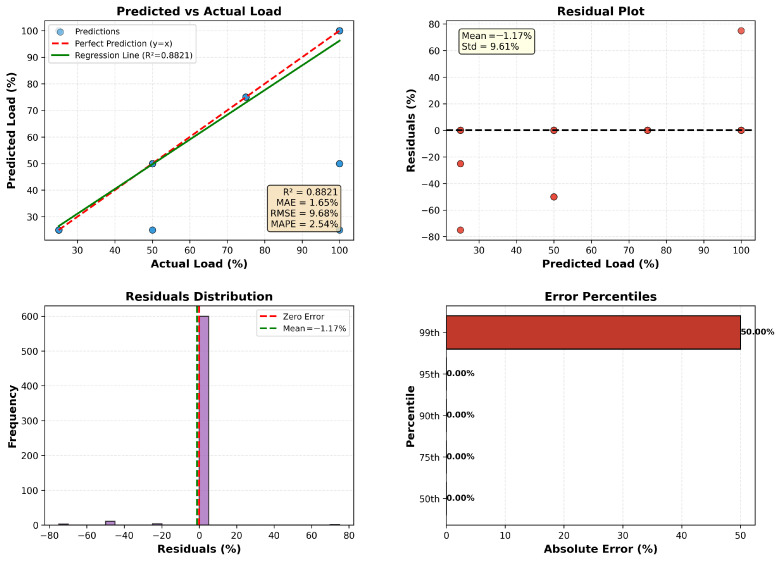
ANN Regression Analysis.

The proposed ANN regression model (Figure 6) demonstrates strong predictive capability for engine load estimation. As shown in the predicted versus actual load plot, the model achieves an $R^{2}$ of approximately 0.882, indicating that it explains a substantial proportion of the variance in the target variable. Error metrics further confirm its accuracy, with a mean absolute error (MAE) of 1.65%, root mean square error (RMSE) of 9.68%, and mean absolute percentage error (MAPE) of 2.54%. The residual analysis reveals a near-zero mean bias (−1.17%) and a standard deviation of 9.61%, suggesting that prediction errors are symmetrically distributed around zero. The residual distribution plot shows that most errors cluster tightly near zero, while the error percentile chart indicates that extreme deviations are rare, with the 99th percentile well within acceptable limits. These results confirm that the ANN model effectively captures non-linear dependencies among engine parameters, outperforming linear and polynomial regression baselines in both accuracy and robustness.

The confusion matrix confirms excellent classification performance, with diagonal dominance and minimal misclassifications. Accuracy per class exceeds 97%, and the 75% load class achieves perfect prediction (100%), indicating strong separability among operating conditions. Minor confusion occurs between the 100% and 50% load classes (approximately 2.67%), likely due to overlapping feature ranges at high torque. The correlation heatmap reveals strong positive relationships between wheel power and torque (r≈0.83) and between wheel power and design power (≈0.74), while engine speed shows a negative correlation with torque (r≈−0.78). These patterns validate the ANN’s ability to exploit multivariate dependencies for accurate load prediction.

**Figure 7:**
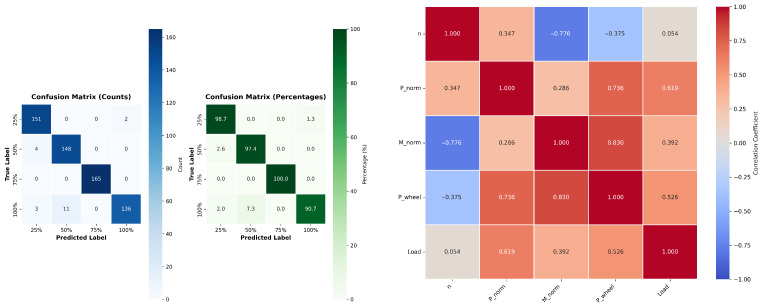
Confusion Matrix and Correlation Heatmap.

With the number of data points being uniformly distributed across categories, we argue that the model is able to clearly distinguish between the different classes (Table 7). As can be seen from the confusion matrix (Figure 7), we find that the model misclassified less than 3% of the sample points in category IV (class 1.0) to category II (class 0.5). This shows that while there is still some similarity between the two classes, the model is still able to perform adequately.

In this matrix (Figure 7), the rows represent the actual classes and the columns represent the predicted classes. Out of 620 observations, 616 were classified correctly, giving an overall accuracy rate of 99.35%. The only misclassification occurred between classes II (likely 0.5) and IV (likely 1.0), where four examples from class IV were labeled as belonging to class II. The confusion rate for class IV is therefore approximately 2.67% (4 out of 150 instances).

The robustness of the model was demonstrated through polynomial regression, with the $R^{2}$ value increasing from 0.8688 for the linear model to 0.9607 for the third-degree model for the $P_{n}$. This indicates a strong non-linear relationship with the predicted load. The confusion matrix shows that the model performed almost perfectly in classifying all four categories: only four out of 620 samples were misclassified, demonstrating the model’s precision and generalization capability.

The data from Table 4 describe the engine’s behavior at its extreme operating points (minimum and maximum) and provide insights for the continuous analysis of its performance. In the context of machine learning, the predictions for the maximum points lean towards overloading the internal combustion engine, which is difficult to achieve due to mechanical limitations, while the predictions for the minimum points lead to a significantly under-revved range of the internal combustion engine, which is also difficult to achieve due to design constraints. Both situations are considered theoretical cases, being impossible to realize.

It is important to note that an uncertainty analysis was performed according to the suggestions included in article [38]. The numerical results presented in this study demonstrate that the proposed model achieves high predictive accuracy under normal operating conditions.

### 4.1. Future Improvements

From a software perspective, the model can be deployed as an API or embedded in existing software platforms for integration with web, mobile, or industrial applications. It is possible to implement even more real-time monitoring (predictive maintenance, anomaly detection, or load forecasting in industrial or energy systems) and scalable deployment of cloud-based applications (to handle large-scale data and concurrent requests).

Nevertheless, from the perspective of artificial intelligence, it supports transfer learning, or more complex decision-making.

In order to improve the proposed model, the following aspects are to be considered:

- The uniform distribution of observations across classes helped stabilize the network. When dealing with imbalanced data, it may be necessary to weight the loss function or apply recalibration techniques.
- The four examples confused between classes II and IV should be examined separately. As they may share characteristics of both classes or have noisy labels, manual analysis or dimensionality reduction could provide insight.
- To further reduce confusion, one could experiment with deeper architectures or regularization techniques, such as dropout or batch normalization. Improving class separability can also be achieved by tuning hyperparameters, increasing the size of the training set, or using data augmentation.
- In addition to accuracy, precision and recall, other measures such as the macro-F1 score or a weighted confusion matrix can reveal the model’s behavior. Continuous performance monitoring is recommended because the confusion matrix offers a concise summary of how the model labels each observation.

The ANN with four inputs $\left(P_{n},\;P_{\mathrm{wheel}},\;M_{n},\;n\right)$, load depends on nonlinear and regime-dependent interactions (e.g., torque-engine speed tradeoffs and drivetrain losses). As a compact multilayer perceptron (MLP) provides the right balance of capacity and efficiency for this mapping:

Nonlinearity Handling. ReLU-based hidden layers implement a piecewise-linear approximation that captures cross-feature interactions and thresholds intrinsic to engine operation. This yields a single differentiable mapping that typically outperforms linear models or simple logistic regressions on multi-class load prediction.

Extensibility. The architecture scales naturally with additional signals (e.g., ambient temperature, EGR rate). Adding features only requires resizing the input layer and retraining; the same network head supports both classification (Softmax) and regression (Linear), avoiding separate pipelines.

Robustness. Dropout and early stopping provide regularization against small-sample overfitting and sensor noise. Using stratified *k*-fold cross-validation (here, k=5) yields stable metrics across operating regimes and reduces variance in performance estimates.

Computational efficiency. For the chosen architecture (Dense (30, ReLU) → Dense (18, ReLU) → output), the parameter count remains small:

$$\begin{array}{cc} \mathrm{Classification}\;(4\;\mathrm{classes}):\; & 4\times 30+30\times 18+18\times 4\;+\;(30+18+4)\;=\;784\;\mathrm{parameters},\\ \mathrm{Regression}\;(\mathrm{scalar}\;\mathrm{output}):\; & 4\times 30+30\times 18+18\times 1\;+\;(30+18+1)\;=\;727\;\mathrm{parameters}. \end{array}$$

This supports sub-millisecond inference on typical embedded targets and straightforward deployment.

### 4.2. Limitations and Future Work

Although the proposed ANN model achieved high accuracy, the training dataset was relatively small and only covered specific operating conditions. This increases the risk of overfitting and may affect generalization to other engine types or environments. Real-time deployment of the model in embedded systems or cloud-based platforms could facilitate predictive maintenance and continuous monitoring in industrial and automotive applications.

Although the methodology was validated on a 1.9-L light-duty diesel engine, it can be adapted to other engine types and powertrain systems if the necessary sensor data and calibration parameters are available.

The results were demonstrated under normal operating conditions and quasi-steady sweeps. However, behavior under richer transients or edge cases was not comprehensively validated.

## 5. Conclusions

On the 2064 sample dataset collected from a 1.9-L TDI engine over four load classes and 1545–4195 rpm, our ANN achieved ≈0.99 accuracy and ≈0.99 macro-F1, with cross-validated regression $R^{2}=0.962\pm 0.052$, and a 99.35% test accuracy confusion matrix indicating strong separability.

Because features and labels in our pipeline align with ECU-available signals and dyno-calibrated load, the trained ANN can be dropped into existing monitoring/calibration workflows with minimal re-instrumentation, offering immediate value for predictive maintenance and calibration trimming.

Based on the collected data and the trained ANN, the following conclusions can be drawn:

- Through machine learning, the efficiency of an internal combustion engine can be determined more easily.
- The internal combustion engine’s useful lifetime can be estimated [39,40].
- By training the artificial neural networks using the method proposed in this paper, other functional parameters of a combustion engine can be added to the model in a similar way, either as input (if they are easy to measure) or as predicted values (if the measurements are missing) and further optimized, e.g., parameters such as compression ratio, injection advance angle, etc.
- The performance of the ANN model can be improved by changing the hyperparameters, such as the number of epochs, the architecture of the network, the learning rate and the batch size.
- Predictions for the maximum points tend to overload the combustion engine, while predictions for the minimum points lead to significant under-revving of the combustion engine.

The machine learning algorithm developed for the internal combustion engine in an automobile can be adapted for use with an electric motor due to its ability to analyze and optimize performance metrics that are fundamental to both types of engines. While internal combustion engines and electric motors have different operating principles, both share common factors such as torque, engine rotational speed, and power output, which are essential inputs in performance and efficiency models. By adjusting certain input parameters and training data, the same algorithmic framework can be applied to electric motors to monitor and enhance energy efficiency, predict maintenance needs, and optimize power delivery in real time.

## Figures and Tables

**Table 1:** Sample Data.

n [RPM]	$P_{n}$ [kW]	$M_{n}$ [Nm]	$P_{\mathrm{wheel}}$ [kW]	Load [-]
1545	10.833	66.957	7.461	0.25
3955	11.793	28.474	0.877	0.25
3910	20.839	50.892	9.975	0.75

**Table 2:** Input Dataset Summary.

Feature	Min	Max	Mean	Std
*n*	1545.00	4195.00	2834.30	747.98
$P_{n}$	8.27	23.80	18.49	3.43
$M_{n}$	28.47	95.13	66.14	16.49
$P_{wheel}$	0.88	15.78	11.15	3.49
Load	0.25	1.00	0.63	0.28

**Table 3:** Hyperparameters for Classification and Regression Models.

Hyperparameter	Classification Model	Regression Model
Input Features	4 ($P_{n}$, $P_{wheel}$, $M_{n}$, *n*)	4 (same)
Hidden Layers	Dense (30, ReLU), Dense (18, ReLU)	Same as classification
Dropout Rates	0.2, 0.1	0.2, 0.1
Output Layer	Dense (4, Softmax)	Dense (1, Linear)
Loss Function	Sparse categorical cross-entropy	Mean squared error (MSE)
Optimizer	Adam (learning rate $10^{-3}$)	Adam (learning rate $10^{-3}$)
Batch Size	10	10
Epochs	100	100
Early Stopping	Patience = 15, min_delta = $10^{-5}$	Same
Regularization	Dropout only	Dropout only
Validation	5-fold stratified cross-validation (mean ± SD)	Same
Random Seed	12	12
Performance Metric	Accuracy ≈0.99, F1 ≈0.99	$R^{2}\approx 0.98$

**Table 4:** Model Predictions.

*n* [rpm]	$P_{n}$ [kW]	$M_{n}$ [Nm]	$P_{\mathrm{wheel}}$ [kW]	$\hat{L}$ [–]
500	5.00	10.00	3.00	0.227
1675	14.19	80.92	10.48	0.346
3970	17.51	42.45	5.99	0.612
1770	17.00	91.75	13.07	0.733
6000	25.00	60.00	10.00	0.999

**Table 5:** Regression Metrics for Predicted Load.

Variable	Coefficient	Intercept	R^2^
$P_{n}$	0.0398	−0.0428	0.8688
$M_{n}$	0.0041	0.3517	0.1832
$P_{wheel}$	0.0428	0.2188	0.3079
*n*	0.0001	0.2510	0.7230

**Table 6:** Polynomial Regression R^2^ Comparison for Predicted Load.

Variable	R^2^ (Degree 2)	R^2^ (Degree 3)
$P_{n}$	0.9021	0.9607
$M_{n}$	0.4683	0.5064
$P_{wheel}$	0.3993	0.4441
*n*	0.7239	0.8149

**Table 7:** Classification Report.

Class	Precision	Recall	F1-Score	Support
0.25	1.00	1.00	1.00	153
0.50	0.97	1.00	0.99	152
0.75	1.00	1.00	1.00	165
1.00	1.00	0.97	0.99	150
Accuracy			0.99	620
Macro avg	0.99	0.99	0.99	620
Weighted avg	0.99	0.99	0.99	620

## Data Availability

The data supporting the findings of this study are available from the source cited in [17].

## Appendix A. ANN Model

**Table A1:** Activation functions and layer computations used in the model.

ReLU activation function	ReLU(*x*) = max(0, *x*)
Softmax function	$\mathrm{Softmax}\left(z_{i}\right)=\frac{e^{z_{i}}}{\sum_{j=1}^{C}e^{z_{j}}},\;i=1,\ldots ,C$
Layer 1 (Dense + ReLU)	$z^{\left(1\right)}=W^{\left(1\right)}x+b^{\left(1\right)},\;a^{\left(1\right)}=\mathrm{ReLU}\;\left(z^{\left(1\right)}\right)$
Layer 2 (Dense + ReLU)	$z^{\left(2\right)}=W^{\left(2\right)}a^{\left(1\right)}+b^{\left(2\right)},\;a^{\left(2\right)}=\mathrm{ReLU}\;\left(z^{\left(2\right)}\right)$
Layer 3 (Dense + ReLU)	$z^{\left(3\right)}=W^{\left(3\right)}a^{\left(2\right)}+b^{\left(3\right)},\;a^{\left(3\right)}=\mathrm{ReLU}\;\left(z^{\left(3\right)}\right)$
Output layer (Dense + Softmax)	$z^{\left(4\right)}=W^{\left(4\right)}a^{\left(3\right)}+b^{\left(4\right)},\;\hat{y}=\mathrm{Softmax}\;\left(z^{\left(4\right)}\right),\;{\hat{y}}_{i}=\frac{e^{z_{i}^{\left(4\right)}}}{\sum_{j=1}^{C}e^{z_{j}^{\left(4\right)}}}$

Softmax function—converts a vector of raw scores $z=[z_{1},z_{2},\ldots ,z_{C}]$ into a probability distribution over *C* classes.

## References

[1] Khan P.W., Byun Y.C. (2024). A review of machine learning techniques for wind turbine’s fault detection, diagnosis, and prognosis. Int. J. Green Energy.

[2] Monieta J., Kasyk L. (2023). Application of Machine Learning to Classify the Technical Condition of Marine Engine Injectors Based on Experimental Vibration Displacement Parameters. Energies.

[3] Solmaz O., Gurbuz H., Karacor M. (2020). Comparison of Artificial Neural Network and Fuzzy Logic Approaches for the Prediction of In-Cylinder Pressure in a Spark Ignition Engine. J. Dyn. Syst. Meas. Control.

[4] Aliramezani M., Koch C.R., Shahbakhti M. (2022). Modeling, diagnostics, optimization, and control of internal combustion engines via modern machine learning techniques: A review and future directions. Prog. Energy Combust. Sci..

[5] Shen Y., Khorasani K. (2020). Hybrid multi-mode machine learning-based fault diagnosis strategies with application to aircraft gas turbine engines. Neural Netw..

[6] Ramachandran E., Krishnaiah R., Venkatesan E.P., Parida S., Dwarshala S.K.R., Khan S.A., Asif M., Linul E. (2023). Prediction of RCCI combustion fueled with CNG and algal biodiesel to sustain efficient diesel engines using machine learning techniques. Case Stud. Therm. Eng..

[7] Liu J., Huang Q., Ulishney C., Dumitrescu C.E. (2021). Machine learning assisted prediction of exhaust gas temperature of a heavy-duty natural gas spark ignition engine. Appl. Energy.

[8] Srinivaas A., Sakthivel N., Nair B.B. (2025). Machine Learning Approaches for Fault Detection in Internal Combustion Engines: A Review and Experimental Investigation. Informatics.

[9] Zhou L., Song Y., Ji W., Wei H. (2022). Machine learning for combustion. Energy AI.

[10] Zheng Z., Lin X., Yang M., He Z., Bao E., Zhang H., Tian Z. (2020). Progress in the application of machine learning in combustion studies. ES Energy Environ..

[11] Badra J.A., Khaled F., Tang M., Pei Y., Kodavasal J., Pal P., Owoyele O., Fuetterer C., Mattia B., Aamir F. (2021). Engine combustion system optimization using computational fluid dynamics and machine learning: A methodological approach. J. Energy Resour. Technol..

[12] Namar M.M., Jahanian O., Koten H. (2022). The Start of Combustion Prediction for Methane-Fueled HCCI Engines: Traditional vs. Machine Learning Methods. Math. Probl. Eng..

[13] Aghbashlo M., Peng W., Tabatabaei M., Kalogirou S.A., Soltanian S., Hosseinzadeh-Bandbafha H., Mahian O., Lam S.S. (2021). Machine learning technology in biodiesel research: A review. Prog. Energy Combust. Sci..

[14] Bhattacharya A., Majumdar P. (2024). Artificial intelligence-machine learning algorithms for the simulation of combustion thermal analysis. Heat Transf. Eng..

[15] Sun X., Xie M., Zhou F., Fu J., Liu J. (2023). Multi-objective optimization for combustion, thermodynamic and emission characteristics of Atkinson cycle engine using tree-based machine learning and the NSGA II algorithm. Fuel.

[16] Chiriac R.L., Chiru A., Boboc R.G., Kurella U. (2021). Advanced Engine Technologies for Turbochargers Solutions. Appl. Sci..

[17] Chiriac R.L., Aldea C., Bocu R. (2025). Dynamometric Measured Parameters for Combustion Engine.

[18] MAHA Maschinenbau Haldenwang GmbH & Co. KG (2023). LPS 3000—Dynamometer for Passenger Cars, Commercial Vehicles and Motorcycles—Original Operating Instructions.

[19] Singh S.K., Singh S., Sehgal A.K. (2016). Impact of Low Viscosity Engine Oil on Performance, Fuel Economy and Emissions of Light Duty Diesel Engine.

[20] Hamza K., Chu K.C., Favetti M., Benoliel P.K., Karanam V., Laberteaux K.P., Tal G. (2021). Comparisons of Real-World Vehicle Energy Efficiency with Dynamometer-Based Ratings and Simulation Models. World Electr. Veh. J..

[21] Harach T., Simonik P., Vrtkova A., Mrovec T., Klein T., Ligori J.J., Koreny M. (2023). Novel Method for Determining Internal Combustion Engine Dysfunctions on Platform as a Service. Sensors.

[22] (2011). Engine Power Test Code—Spark Ignition and Compression Ignition Engines.

[23] Willcox M.A., Cleeves J.M., Jackson S., Hawkes M., Raimond J. (2012). Indicated Cycle Efficiency Improvements of a 4-Stroke, High Compression Ratio, SI, Opposed-Piston, Sleeve-Valve Engine Using Highly Delayed Spark Timing for Knock Mitigation.

[24] Ko J., Ko S., Son H., Yoo B., Cheon J., Kim H. (2014). Development of brake system and regenerative braking cooperative control algorithm for automatic-transmission-based hybrid electric vehicles. IEEE Trans. Veh. Technol..

[25] Koch A., Nicoletti L., Herrmann T., Lienkamp M. (2022). Implementation and analyses of an eco-driving algorithm for different battery electric powertrain topologies based on a split loss integration approach. Energies.

[26] Lee J.W., Kim S.C., Oh J., Chung W.J., Han H.W., Kim J.T., Park Y.J. (2019). Engine speed control system for improving the fuel efficiency of agricultural tractors for plowing operations. Appl. Sci..

[27] Reitz R.D., Ogawa H., Payri R., Fansler T., Kokjohn S., Moriyoshi Y., Agarwal A., Arcoumanis D., Assanis D., Bae C. (2020). IJER editorial: The future of the internal combustion engine. Int. J. Engine Res..

[28] Korendyasev G., Salamandra K., Tyves L. (2019). Analysis of gearshift processes in an automatic transmission at low vehicle speeds. Vibroeng. Procedia.

[29] Zhang Z., Liu H., Yue Z., Li Y., Liang H., Kong X., Zheng Z., Yao M. (2023). Effects of intake high-pressure compressed air on thermal-work conversion in a stationary diesel engine. Int. J. Green Energy.

[30] Yoo H., Park B.Y., Cho H., Park J. (2019). Performance optimization of a diesel engine with a two-stage turbocharging system and dual-loop EGR using multi-objective Pareto optimization based on diesel cycle simulation. Energies.

[31] Dahham R.Y., Wei H., Zhang R., Li J., Shu G., Pan J. (2025). Numerical study for the comparison between direct dual-fuel stratification and reactivity-controlled compression ignition of ammonia-based engines. Appl. Therm. Eng..

[32] Novotnỳ P., Vacula J., Hrabovskỳ J. (2021). Solution strategy for increasing the efficiency of turbochargers by reducing energy losses in the lubrication system. Energy.

[33] Sciarretta A., Vahidi A. (2020). Energy-Efficient Driving of Road Vehicles.

[34] Chojnowski J., Karczewski M. (2022). Influence of the working parameters of the chassis dynamometer on the assessment of tuning of dual-fuel systems. Energies.

[35] Mihalič F., Truntič M., Hren A. (2022). Hardware-in-the-loop simulations: A historical overview of engineering challenges. Electronics.

[36] Lamberg K. (2006). Model-based testing of automotive electronics. Proceedings of the Design Automation & Test in Europe Conference.

[37] Abboush M., Bamal D., Knieke C., Rausch A. (2022). Hardware-in-the-loop-based real-time fault injection framework for dynamic behavior analysis of automotive software systems. Sensors.

[38] Habib G. (2020). The effect of H2 purity on the combustion, performance, emissions and energy costs in an SI engine. Therm. Sci..

[39] Yang R., Yan Y., Sun X., Wang Q., Zhang Y., Fu J., Liu Z. (2022). An artificial neural network model to predict efficiency and emissions of a gasoline engine. Processes.

[40] Yang R., Xie T., Liu Z. (2022). The application of machine learning methods to predict the power output of internal combustion engines. Energies.

